# Correlation Analysis of the Tinnitus Handicap Inventory and Distress Network in Chronic Tinnitus: An EEG Study

**DOI:** 10.32598/bcn.9.10.215

**Published:** 2019-09-01

**Authors:** Samer Mohsen, Saeid Mahmoudian, Saeed Talbian, Akram Pourbakht

**Affiliations:** 1. Department of Otolaryngology, School of Medicine, Damascus University, Damascus, Syria.; 2. ENT and Head & Neck Research Center, Iran University of Medical Sciences, Tehran, Iran.; 3. School of Rehabilitation, Tehran University of Medical Sciences, Tehran, Iran.; 4. Department of Audiology, School of Rehabilitation Sciences, Iran University of Medical Sciences, Tehran, Iran.

**Keywords:** Tinnitus, Tinnitus-related distress, Tinnitus Handicap Inventory (THI), Correlation, Electroencephalography, Functional connectivity

## Abstract

**Introduction:**

Tinnitus is a common disorder with a considerable amount of distress that affects the patient‘s daily life. No objective tools were approved for measuring tinnitus distress. It can be estimated only by subjective scales and questionnaires, albeit, the Electroencephalography (EEG) studies have reported some alterations regarding tinnitus distress network. This study aimed to investigate the correlation between Tinnitus Handicap Inventory (THI) and the recorded EEG data.

**Methods:**

A total of 33 chronic tinnitus cases (9 females) with the mean age of 42.67 years were recruited. Their THI scores were collected, and a 3-minute EEG recorded with eye closed at resting-state. The correlation analysis was performed on THI scores and the current density in the selected Region of Interests (ROIs) concerning the distress network for the eight frequency bands. The patients grouped depending on the THI cutoff point of 56 into low and high THI groups, and then the groups were compared for source analysis and functional connectivity between ROIs using standardized low-resolution brain electromagnetic tomography.

**Results:**

A significant positive correlation was seen between THI scores and the electrical activity in the Anterior Cingulate Cortex (ACC), the prefrontal cortex, and the parahippocampus for an alpha band (P<0.05) and in the ACC for beta (P<0.01). Source analysis showed significant differences with increased activity in the high THI group for alpha, beta and gamma bands. Functional connectivity was also elevated in the high THI group between the ROIs in alpha and beta bands.

**Conclusion:**

THI can be a useful tool for measuring tinnitus distress, and it has a high correlation with EEG data.

## Highlights

- THI score is a suitable tool for indicating the amount of tinnitus distress;
- THI score has a good correlation with tinnitus distress activity;
- sLORETA analysis showed important findings in tinnitus distress according to THI.

## Plain Language Summary

Tinnitus is a common disorder with a considerable amount of distress that affects the person‘s daily life. No objective tools were approved for measuring tinnitus distress. It can be estimated only by subjective scales and questionnaires, albeit, the Electroencephalography (EEG) studies have reported some alterations regarding tinnitus distress network. The aim of this study was to investigate the correlation between Tinnitus Handicap Inventory (THI) and the recorded EEG data. The study results showed a significant positive correlation between THI scores and the electrical activity in the distress network activity in the brain. Important differences were noticed between the high and low distress groups. This means that THI can be a used as a tool for measuring tinnitus distress with a high correlation to EEG data.

## 1. Introduction

Hearing a sound(s) in the ears or the head without any external source is called tinnitus (Jastreboff, 1990). This phantom perception or phenomenon can be one of the most stressful experiences for a person (Heller, 2003). Looking at tinnitus epidemiology, we can expect that approximately 10%–15% of the society members are suffering from tinnitus (Sanchez, 2004; Bhatt, Lin, & Bhattacharyya, 2016). The problem gets more prevalent and severe with increasing age (Sindhusake et al., 2003). It can affect daily activities, sleep, concentration and cause functional and mood disorders (Henry, Dennis, & Schechter, 2005). Earlier reports indicated that 6%–25% of the tinnitus sufferer declared some reduction of their quality of life (Baguley, 2002) with the most debilitating degrees occurring in 2%–4% of the population (Axelsson & Ringdahl, 1989). As a problem with high prevalence and substantial distress, tinnitus has become one of the popular subjects of medical, behavioral and neuroscience studies (Landgrebe et al., 2012).

Objective tools cannot measure tinnitus distress since it is a subjective feeling with no correlation to the psychoacoustic properties of the perceived sound such as matched loudness, pitch, and hearing loss or even with the demographic characteristics of the patients (Andersson, 2003; Hallam, Rachman, & Hinchcliffe, 1984; Nooruzian et al., 2017). Therefore, several subjective scales and self-reported questionnaires have been established for measuring tinnitus distress and intensity depending on its impact on daily life and many other features.

The visual analog scale is one of the easy to use tools for estimating tinnitus loudness and distress through giving a degree from 0 to 10 depending on the patient’s feelings (Price, McGrath, Rafii, & Buckingham, 1983).

Tinnitus Handicap Inventory (THI), alongside some other questionnaires, is a well-established and widely-used tinnitus measure for clinical and research purposes. THI has a high validity (r>0.80) and test-retest reliability (95% of the observed differences falling within ±2 SD), as well as good adequacy for evaluating treatment outcome (Newman, Sandridge, & Jacobson, 1998). Three principle dimensions are covered in these items: functional, emotional, and catastrophic subscales (Kleinstäuber, Frank, & Weise, 2015; Newman, Jacobson, & Spitzer, 1996).

The THI has been translated and validated in many languages, so it became an international tool for assessing the tinnitus-related handicap and its impact on daily living, especially in busy clinics. The Persian version or THI-P was translated and standardized by Mahmoudian, Shahmiri, Rouzbahani, Jafari, Reza Keyhani, (2011), and its psychometric properties were validated by Jalali, Soleimani, Fallahi, Aghajanpour, & Elahi, (2015).

Depending on the total score of THI, tinnitus can be classified into five grades, from slight to catastrophic handicaps. These five grades can also be divided into two categories of T1 from 0–56 points and T2 from 57–100 points based on the tinnitus impact on daily life, that is the patient can do everyday activities in grades 1–3 and in the higher grades he cannot (McCombe et al., 2001; Takahashi et al., 2017).

Neuroimaging studies have almost succeeded in unraveling the neural correlates of tinnitus (Husain, 2016; Lanting, de Kleine, & van Dijk, 2009; Schmidt, Zimmerman, Bido Medina, Carpenter-Thompson, & Husain, 2018). In other words, the involvement of auditory and non-auditory areas in generating and perception of tinnitus, and its accompanying distress have been well explained (De Ridder et al., 2014). Encephalography research on tinnitus patients revealed several alterations in the oscillatory power of the frequency bands of brain function. The decreased alpha power in the auditory cortex has been substituted by increased theta power, and an increase of gamma power in the contralateral cortex was reported by some studies (Llinás, Ribary, Jeanmonod, Kronberg, & Mitra, 1999; Lorenz, Müller, Schlee, Hartmann, & Weisz, 2009; Weisz et al., 2007).

Regarding tinnitus distress, a recent EEG study using standardized low-resolution brain electromagnetic tomography (sLORETA) source localization highlighted the relationship between tinnitus distress and increasing of beta-band activity in the Anterior Cingulate Cortex (ACC) and the amount of distress was related to the alpha band activity in several brain areas that form the distress network (Vanneste et al., 2010a). Several EEG and fMRI studies on tinnitus patients corroborated the increased activity in distress network, which consists of the amygdala (Chen et al., 2017), ACC (Vanneste, Joos, Ost, & De Ridder, 2018), insula (van der Loo, Congedo, Vanneste, De Heyning, & De Ridder, 2011), and parahippocampus (De Ridder et al., 2006). These hubs are interconnected and overlapped functionally with tinnitus network (De Ridder, 2014). Furthermore, some other reports revealed the increased functional connectivity between the precuneus and the Orbitofrontal Cortex (OFC) and Dorsolateral Prefrontal Cortex (DLPFC) (Schlee et al., 2009; Schmidt, Carpenter-Thompson, & Husain, 2017) in beta and gamma bands. These frontal areas showed some laterality for emotional processing, that is, the right OFC and DLPFC are related to distress network while the left side is linked to depression network (Joos, Vanneste, & De Ridder, 2012).

As previously stated, EEG studies have yielded valuable data about tinnitus-related distress neural correlate; however, there are some conflicting data due to tinnitus heterogeneity and also for the high variability in tinnitus EEG studies methodologies (Møller, 2007). To use these data in diagnostic and therapeutic aspects, they should be correlated with subjective data. So far, several studies have assigned good correlations between the electrical activity at distress network and the amount of distress calculated by Tinnitus Questionnaire (TQ) (Song, De Ridder, Schlee, Van de Heyning, & Vanneste, 2013a). Such data can be beneficial, especially for following the treatment effects and also make right decisions in treatments like neurofeedback and neuromodulation (Moossavi & Mohsen, 2016).

THI as a measure of tinnitus handicap could show good correlations with other self-reported and -rating psychological scales like the visual analog scale of tinnitus annoyance (Figueiredo, de Azevedo, & de Mello Oliveira, 2009), tinnitus acceptance (Westin, Hayes, & Anderson, 2008), and tinnitus-related distress (Monzani et al., 2008; Salviati et al., 2013). Thus, we can assume that THI can be a gold standard for assessing tinnitus distress if it has a good correlation with the previously mentioned EEG or neuroimaging data of distress network. The main goal of this study is to investigate the relationship between the THI scores in a homogenous tinnitus group with the recorded EEG electrical activity after controlling the other important confounding factor i.e., tinnitus-related loudness. Then, we will use the THI cutoff point of 56 to have two groups with high and low THI scores and compare them with regard to distress network electrical activity.

## 2. Methods

### 2.1. Study participants

Thirty-three right-handed patients (9 females) with non-pulsatile chronic tinnitus (duration more than 6 months) were recruited in this study. Their Mean±SD age was 42.67±10.95 years. All patients had no history of Meniere’s disease, otosclerosis, or any other middle ear problems, no neurological disorders such as head trauma, and brain tumors. Those who were receiving medications for mental or psychological disorders were excluded. To get a more homogenous group, all patients were given the validated Persian version of Hospital Anxiety and Depression Scale (HADS) (Montazeri, Vahdaninia, Ebrahimi, & Jarvandi, 2003) and those with a score of 21 or less (less than 11 for either depression and anxiety subscales, indicating no depression or anxiety disorders) were included in the study (Snaith, 2003).

All patients were sent to the auditory clinic for the psychoacoustic measurement of their tinnitus. The behavioral pure-tone audiometry thresholds levels were less than 20 dB HL in octave frequencies of 250–2000 Hz and not more than 40 dB HL in the higher frequencies (4000–8000 Hz) which refer to normal hearing or mild hearing loss in high frequencies and higher hearing levels were excluded. All types of tinnitus were accepted with no limitations regarding the psychoacoustic aspects and laterality (Lim, Lu, Koh, & Eng, 2010). This study is a part of a randomized clinical trial registered at Iranian Registry of Clinical Trials (Identifier: 20586 30/6/2017) and was approved by the Ethics Committee of Iran University of Medical Sciences. After explaining research methodology, written informed consent was taken from the subjects.

#### 2.1.1. Tinnitus handicap inventory questionnaire and grouping

All patients filled in the items of the THI-P (Mahmoudian et al., 2011). It contains 25 items with three choices (Yes, No, and sometimes) and a total score range of 0–100. The total scores were computed, and the patients were allocated into two groups depending on their achieved scores: the low and high THI group with scores less and more than 56, respectively. As previously stated, this cutoff point was used for grading tinnitus handicap as compensated or decompensated through its effects on the patient’s daily activity (McCombe et al., 2001). Furthermore, the Visual Analogue Scale (VAS) was used to assess the tinnitus Loudness (VAS-L) and Annoyance (VAS-A). Each patient was asked to give a degree from 0–10 depending on the intensity of his problem.

#### 2.1.2. EEG data collection

EEG recordings were obtained using a 64-channel Brain Quick LTM (Micromed, Italy) in a shielded room against sounds and electromagnetic signals. Each participant sat upright in a comfortable chair and using pillows to reduce the muscles’ contraction. The actual recording time lasted 3 minutes with eyes closed. The recordings were sampled using 29 electrodes placed on FP1, FPz, FP2, F7, F3, Fz, F4, F8, FT7, FC3, FCz, FC4, FT8, T7, C3, Cz, C4, T8, TP7, CP3, CPz, CP4, TP8, P3, P4, POz, and Oz according to the international 10–20 system (Jasper, 1958) referenced to the tip of nose and the ground electrode placed on the forehead. The electro-oculogram was recorded using two electrodes placed below and near the outer canthus of the left eye. Electrodes impedances were checked and kept below 10 kΩ. The online sampling rate was 1024 with a band-pass filter of 0.4–200 Hz.

EEG analysis was performed offline in the MATLAB v. 8.4, R2014b environment. The obtained data were band-pass filtered (Finite impulse response, FIR filter) to 1.5–70 Hz and subsequently transposed into EEG Lab software (Delorme & Makeig, 2004). Using EEG Lab, the data were plotted and inspected for manual artifact rejection; then, an independent component analysis ICA was conducted to exclude remaining artifacts resulting from eye blinks, eye movement, teeth clenching, and electrocardiography from the EEG stream. After that, the average Fourier cross-spectral matrices were computed for the eight frequency bands: delta (2–3.5 Hz), theta (4–7.5 Hz), alpha1 (8–10 Hz), alpha2 (10–12 Hz), beta1 (13–18 Hz), beta2 (18.5–21 Hz), beta3 (21.5–30 Hz), and gamma (30.5–44 Hz). For the later stages, data were resampled to 256 Hz and referenced again to the standard average reference.

#### 2.1.3. Source localization analysis

For source analysis, we used sLORETA software V. 20081104 provided by the KEY Institute for Brain-Mind Research (University Hospital of Psychiatry, Zurich, Switzerland; http://www.uzh.ch/keyinst/NewLORETA/LORETA01.htm). sLORETA uses algorithms to identify the intracerebral electrical sources that generated the scalp-recorded activity through conventional EEG in each of the 8 frequency bands (Pascual-Marqui, 2002). It calculates the current density (A/m2) from the electric neuronal activity in a spatial space consists of 6239 voxels (voxel size 5*5*5 mm3) without considering a predefined number of active sources (Pascual-Marqui, Esslen, Kochi, & Lehmann, 2002; Wagner, Fuchs, & Kastner, 2004).

The sLORETA solution space is limited to cortical gray matter and both hippocampi and amygdala, which defined by the digitized Montreal Neurological Institute (MNI) 152 template (Fuchs, Kastner, Wagner, Hawes, & Ebersole, 2002; Mazziotta et al., 2001). sLORETA imaging analysis has received significant validation from studies combining this technique with other source localization methods such as magnetic resonance imaging MRI and fMRI (Mulert et al., 2004; Vitacco, Brandeis, Pascual-Marqui, & Martin, 2002; Worrell et al., 2000), and also PET scan (Positron Emission Tomography) (Dierks et al., 2000; Zumsteg, Wennberg, Treyer, Buck, & Wieser, 2005).

#### 2.1.4. Functional connectivity analysis

Coherence (linear dependence) and phase synchronization (non-linear dependence) over the eight predefined frequency bands were applied using the sLORETA connectivity toolbox. This technique explores the similarity between the time-varying signals recorded from two regions. It was refined by Pascual Marqui to remove the artifacts caused by the volume conduction and spatial resolution of the instantaneous non-physiological contaminating signals (Pascual-Marqui, 2007a, 2007b). These measures are positive and become zero when there is no dependence.

Twenty-eight ROIs were selected depending on the previous literature on tinnitus (De Ridder et al., 2014; Vanneste et al., 2010b; Weisz et al., 2007). Each ROI composed of a single voxel that is the nearest one to the center of the region with a 5 mm radius around the centroid voxel. Table 1 demonstrates the 28 selected ROIs for this study.

**Table 1.** Twenty-eight regions of interest and their MNI coordinates

**Region of Interest**	**BA**	**Centroid Voxel**		

		**X-MNI**	**Y-MNI**	**Z-MNI**
Auditory cortices AC	40L & 41L	−55	−25	10
	40R & 41R	55	−25	10
	22L	−55	−25	5
	22R	55	−20	5
	21L	−60	−20	−15
	21R	60	−15	−15
Insula	13L	−40	−10	10
	13R	40	−5	10
Dorsal anterior cingulate cortex dACC	24L	−5	0	35
	24R	5	0	35
Pregenual anterior cingulate pgACC	32L	−5	30	20
	32R	5	30	20
Subgenual anterior cingulate cortex sgACC	25L	−10	20	−15
	25R	5	15	−15
Posterior cingulate cortex PCC	31L	−10	−50	30
	31R	10	−50	35
Para-hippocampal cortex	27L	−20	−35	−5
	27R	20	−35	−5
	29L	−5	−50	5
	29R	5	−50	5
OFC	10&11L	−20	50	0
	10&11R	20	50	0
DLPFC	9L	−30	30	35
	9R	30	30	35
	46L	−45	35	20
	46R	45	35	20
Precuneus	7L	−20	−65	50
	7R	15	−65	50

BA: Brodmann Area; L: left; R: right; MNI: Montreal Neurological Institute

#### 2.1.5. Region of interest analysis

The log-transformed electrical current density (μA/mm2) was computed for the 28 ROIs, differences in activity between the two THI groups were statistically assessed using nonparametric permutation test SnPM using sLORETA statistic toolbox. About 5000 randomizations were considered which, account for the voxelby-voxel comparisons. A paired Student t-test was used to compare maps using the sLORETA (this method applies a collection of tests for all voxels and all frequency bands); a threshold of P<0.05 was accepted for significant differences, i.e. voxels that had an activity threshold more than the threshold of defined P-value, were detected and reported.

### 2.2. Statistical analysis

Statistical analyses for demographic and tinnitus characteristics were performed using the SPSS V. 19.0 (SPSS Inc., Chicago, IL). First, the Kolmogorov-Smirnov test was used to investigate the normal distribution of data in all variables. Then, the age, tinnitus duration, matched loudness, etc., were compared between the two groups using the Independent t-test and Mann-Whitney test depending on the normal distribution of the data (Table 2). Differences in gender, tinnitus type, and laterality distribution were tested using the Chi-square (χ^2^) test.

**Table 2.** Population statistics and tinnitus characteristics

**Variables**	**Low THI Group n=17**	**High THI Group n=16**	**P**
Age (y), Mean±SD	44.76±10.65	40.44±11.15	0.18
Male/Female	13/4	11/5	0.86
Tinnitus type Tonal/NBN^*^	13/4	13/3	0.68
Tinnitus laterality Left/Right/both ears	6/5/6	5/4/7	0.81
Tinnitus duration (y), Mean±SD	5.44±4.9	5.21±5.1	0.98
Matched-loudness, Mean±SD	5.82±2.5	6.69±3	0.46
Total THI score, Mean±SD	42.24±5.51	67.13±6.36	<0.01
VAS-L score, Mean±SD	5.9±1.3	7.9±1.5	<0.01
VAS-A score, Mean±SD	5.8±1.9	8.34±1.5	<0.01

* NBN: Narrow-Band Noise

For correlation analysis, we used the Pearson test or its non-parametric equivalent (Spearman’s rho) to measure the correlation degree between the THI scores and the ROIs. For this test, we selected the regions that had significant differences in any frequency band, as previously stated in the region of interest analysis. The ROIs selected are the left and right auditory cortices (A1 A2), the left and right OFC, the left and right DLPFC, the left and right ACC and the parahippocampus separately for the alpha, beta and gamma frequency bands. To measure the strength of the relationship between THI and the distress network (current densities at ROIs), we should control another confounding variable, which is the tinnitus loudness measured by VAS-L. For that, we used the partial correlation (Anderson, 1954), which measures the degree of relationship after removing the effect of other variables. Using this method, we can determine the substantive amount of source analyzed current density related to tinnitus distress not to its intensity (measured by VAS-L). It should be said that we used the individual THI scores, not the THI grading to have a continuous variable that can be correlated to a specific ROI activity. Lastly, to confirm the previously obtained results, we conducted independent t-test or its non-parametric equivalent to compare the two groups for the significant ROIs current densities. In this test all voxels were averaged for each selected ROI.

## 3. Results

Table 2 presents the male-female ratio, age, hearing level, tinnitus type, duration, and laterality. There were no significant differences in age, gender, and tinnitus duration between the two groups (All P>0.18).

### 3.1. Correlation between tinnitus handicap inventory and region of interests’ current density

The correlation analysis showed a significant (P<0.05) positive correlation between the total score of THI and the log-transformed current density of the left and right insula (BA13), ACC (BA24,25,32), PCC (BA31), parahippocampus (PHC), as well as, the right OFC (BA10,11) and right DLPFC (BA9) in the alpha band. After controlling the VAS-L score, a significant positive partial correlation (P<0.05) was kept for the right dACC, PCC, PHC, and right DLPFC (Table 3a). Besides, significant (P<0.05) positive correlations were seen in beta band (β1: 13–18 Hz, β2: 18.5–21 Hz) of auditory cortex (left and right BA21, 22), left insula, dACC, sgACC, PHC right DLPFC. After controlling for VAS-L only the dACC had a significant (P<0.01) positive partial correlation with THI (Table 3b). Regarding the gamma band, the analysis revealed a significant (P<0.05) positive relationship between the THI and the current density of the left auditory cortex (BA 21, 22), the left insula, PHC, left OFC and left DLPFC. This significance was lost in the partial correlation (Table 3c).

**Table 3.** Significant correlations and partial correlations for the THI and regions of interests

**Frequency Band**	**ROI-Brodmann Area**	**THI**	**THI Controlled for VAS-L**
a-alpha	13L insula	0.61^**^	0.32
	24R dACC	0.4^*^	0.35^*^
	25R sgACC	0.38^*^	0.15
	32R pgACC	0.51^**^	0.23
	31R PCC	0.39^*^	0.4^*^
	27L PHC	0.47^**^	0.39^*^
	11R OFC	0.8^**^	0.35
	46R DLPFC	0.8^**^	0.34^*^
b-Beta1	21L A2	0.47^**^	0.25
	22L A2	0.49^**^	0.25
	13L Insula	0.48^**^	0.13
	24L dACC	0.84^**^	0.80^**^
	24R dACC	0.82^**^	0.75^**^
	25L sgACC	0.78^**^	0.28
	27L PHC	0.45^**^	0.09
	9R DLPFC	0.77^**^	0.22
c-Gamma	21L A2	0.5^**^	0.26
	22L A2	0.48^**^	0.26
	13L Insula	0.47^**^	0.13
	27L PHC	0.47^**^	0.16
	11L OFC	0.4^*^	0.1
	9L DLPFC	0.38^*^	0.47

A2: Secondary auditory cortex

* P<0.05

### 3.2. High versus low tinnitus handicap inventory groups

#### 3.2.1. Source localization analysis

**Figure 1:**
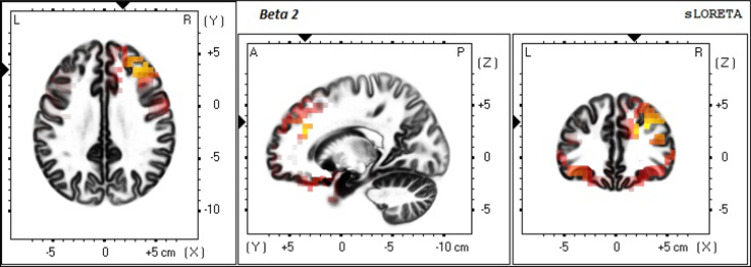
sLORETA contrast analysis between the low and high THI groups Compared to the low THI group, the high THI group revealed an increased activity in the right DLPFC for alpha2, beta1, beta2, and beta3 (only beta2 was showed). Also, there is higher activity in the left DLPFC in gamma band in the high THI group. L: Left; R: Right

**Figure 2.**
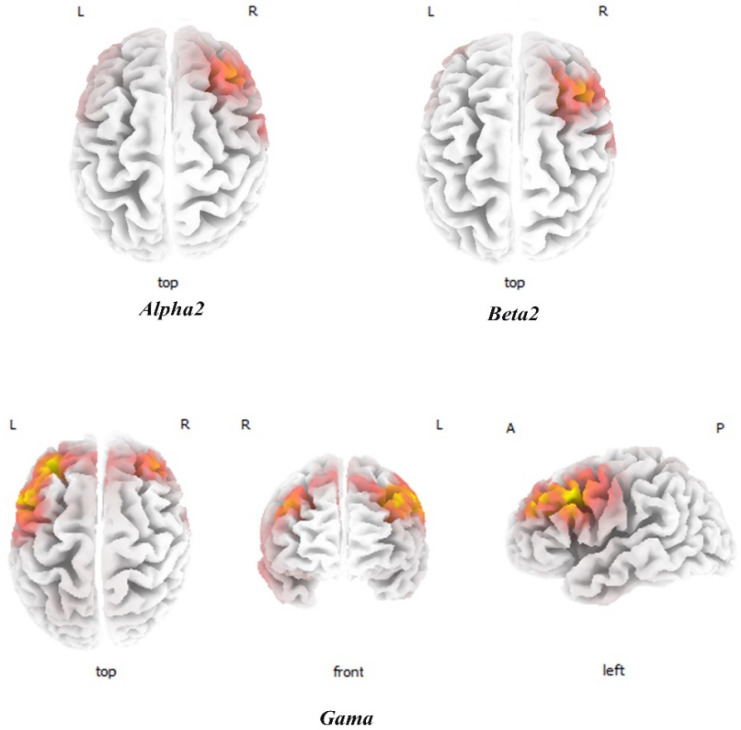
The slice view of sLORETA contrast analysis between low and high THI groups The high THI group showed higher current density values compared to the low THI group in the right DLPFC, right OFC, dACC, and left and right auditory cortex (BA21) for beta2 band. The red color refers to the higher current density (positive values in t-test). The three slices are: axial, sagittal and coronal from left to right respectively. L: Left; R: Right; A: Anterior; P: Posterior

Compared to the low THI group, the high THI group showed increased activity in the right DLPFC for alpha2, beta1, and beta2 and beta3 frequency bands. Also, increased activity was found in the left DLPFC for gamma band (Figure 1). Also, another increased activity was seen in the right OFC, dACC, and right and left auditory cortex (BA21) for beta1 and beta2 bands (Figure 2).

### 3.3. The region of interest analysis

For the area found to be more activated in the source localization comparison, a t-test was performed. The current density among all voxels in the ROI was averaged and compared between the two independent THI groups. Significant differences were noticed in the right OFC and right DLPFC for the alpha band (P<0.01). For the beta band, significant differences were found in the dACC (P<0.01), the right DLPFC (P<0.01), but not for the auditory cortex (P=0.42) and right OFC (P=0.54). Also, the test results revealed a significant difference for gamma band in the left DLPFC (P<0.05) but not in the auditory cortex (P=0.41).

### 3.4. Functional connectivity analysis

The functional connectivity analysis revealed significant differences between the high and low THI groups regarding the alpha and beta bands.

### 3.5. Coherence connectivity

**Figure 3.**
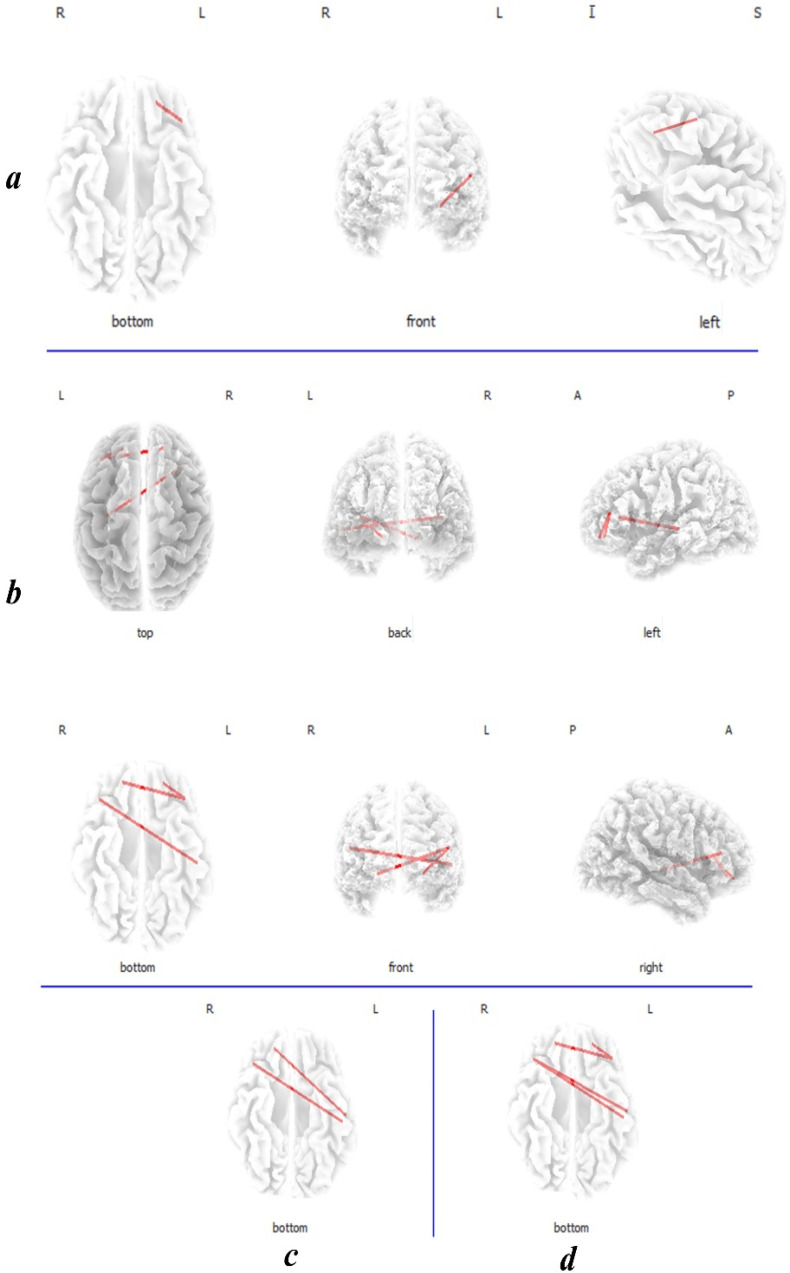
Coherence connectivity contrast analysis between the low THI group and the high THI group a. The high THI group showed increased functional connectivity between the left OFC and left DLPFC in alpha1; b. between the left auditory cortex (BA21) and the right DLPFC BA9, the left DLPFC and the bilateral OFC in beta 2; c. also, an increased functional connectivity with a marginal significance (P<0.1) was noticed between the auditory cortex (BA21, 22) and the right DLPFC and OFC in alpha2; d. between the auditory cortex (BA21, 22) and the right DLPFC in beta3. L: Left; R: Right; A: Anterior; P: Posterior; I: Inferior; S: Superior

The high THI group showed increased coherence functional connectivity between left OFC and left DLPFC in the alpha1 band (Figure 3a). In addition, the high THI group demonstrated increased connectivity between the left auditory cortex (BA21) and the right DLPFC BA9, and between the left DLPFC and the bilateral OFC in beta1 and beta2 frequency bands (Figure 3b). All these differences were significant (t>3.94, P<0.05). Furthermore, increased functional connectivity with a marginal significance (t=3.84 P<0.1) was noticed for alpha2 band between the auditory cortex (BA21, 22) and the right DLPFC and OFC (Figure 3c). Also for beta3 band between the auditory cortex (BA21, 22) and the right DLPFC (Figure 3d).

### 3.6. Lagged phase synchronization

**Figure 4.**
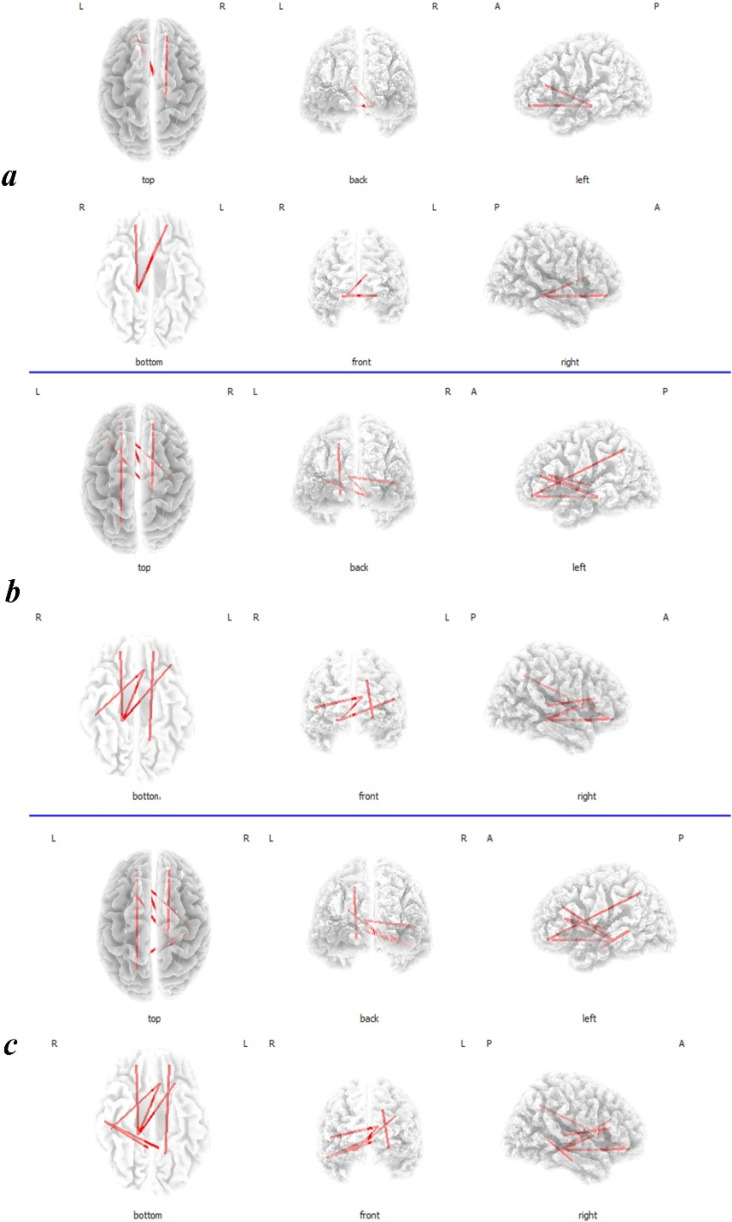
The analysis of lagged phase synchronization connectivity contrast between the low THI group and the high THI group a. The high THI group showed increased functional connectivity between the right parahippocampus and the bilateral OFC and the dACC in alpha2; b. between the parahippocampus and the left DLPFC, right OFC and dACC, between the right auditory cortex (BA22) and the dACC, and also between the left precuneus and the left OFC in beta2; c. Moreover, increased functional connectivity is seen between the right auditory cortex (BA22) and the bilateral PCC in the range frequency of beta3 band, in addition to the previously mentioned connections in beta2. L: Left; R: Right; A: Anterior; P: Posterior

The high THI group showed a significant (t>4.24 P<0.05) increased lagged phase synchronization functional connectivity between the right parahippocampus and the bilateral OFC and the dACC in the alpha2 band (Figure 4a), and also between the parahippocampus and the left DLPFC, right OFC and dACC in the beta1 and beta2 frequency bands (Figure 4b). Besides, another significant increased functional connectivity was noticed between the right auditory cortex (BA22) and the dACC and also, between the left precuneus and the left OFC in the beta2 and beta3 frequency bands. Moreover, a significant increased functional connectivity was detected between the right auditory cortex (BA22) and the bilateral PCC in the range frequency of beta3 band (Figure 4c).

## 4. Discussion

The results of this study revealed that THI questionnaire scores had a good correlation with some EEG features in patients with chronic tinnitus. Grouping tinnitus patients sample using the THI score yielded noticeable differences between the two groups concerning distress network; such results cannot be referred either to differences in the demographic properties of the population or to the tinnitus intensity.

THI scores had a positive relationship with the electrical activity in the brain regions related to the distress network, including the dACC, OFC, DLPFC, PHC, and the insula (Langguth et al., 2012; Leaver et al., 2012; Vanneste et al., 2010b). Likewise, there was a remarkable increased functional connectivity between the previously mentioned hubs and the secondary auditory cortex in the high THI tinnitus group compared to the low THI group.

**Figure 5.**
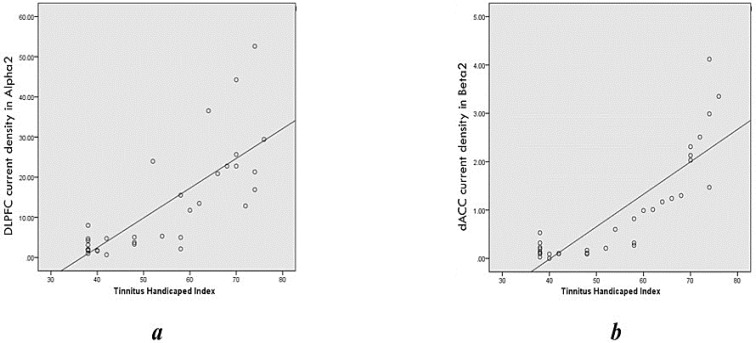
Scatter plots of THI (n=33) A. The current density of DLPFC in alpha2 frequency band; B. The dACC in beta2 frequency band

As mentioned above, our study on THI and resting-state EEG data revealed a significant correlation between the questionnaire scores and the brain activity in the brain areas known recently as the neural substrates of distress. For example, our results showed a positive correlation in an alpha frequency band between the THI and the dACC, PCC, insula, PHC, OFC, and DLPFC; these areas were identified by Vanneste et al. as the processing centers of tinnitus distress (Vanneste et al., 2010b). Other studies have linked the amount of tinnitus-related distress to the alpha band activity in these regions (Ridder, Vanneste, & Congedo, 2011), and that accords with our results of positive correlation (Figure 5a). Moving to the beta frequency band, we found a highly significant positive relationship between THI and the electrical activity of dACC, even after controlling for the tinnitus intensity.

This result is in coherence with other’s findings (Joos et al., 2012; Meyer et al., 2017). The dorsal ACC function was linked to the emotional processing network, and it is involved in the adverse effects caused by tinnitus and similar disorders like chronic pain (Boggio, Zaghi, & Fregni, 2009; Price, 2000) and also the post-traumatic stress disorder (Begić, Hotujac, & Jokić-Begić, 2001). Different neuroimaging studies, such as fMRI studies (Anand et al., 2005) and PET scan studies (Kennedy, Javanmard, & Vaccarino, 1997), also reported similar findings. Moreover, another study has reported that the higher activity of the dACC predicts the higher level of the tinnitus distress the patients feel (Song, De Ridder, Schlee, Van de Heyning, & Vanneste, 2013b). Combining that with our findings, we may claim: the higher activity of the dACC causes a higher amount of distress reflected by the higher scores of THI (Figure 5b).

We used partial correlation in our analysis. Our intent was to control the relationship between THI and EEG data for another important tinnitus aspect like tinnitus intensity that seems to be beneficial. Some of the correlated regions like the auditory cortex (BA21, BA22) and the PHC and the insular cortex in the three frequency bands and the left DLPFC in the gamma band have lost their significance after controlling VAS-L. This is a desired and accepted result, since the network that connected those areas (the insula, PHC, and DLPFC) are more involved in attentional and salience tasks, and they code for tinnitus intensity more than its related distress (Husain, 2016; Husain, Akrofi, Carpenter-Thompson, & Schmidt, 2015; Mirz, Gjedde, Sødkilde-Jrgensen, & Pedersen, 2000).

In our methodology, we divided the patients into two groups depending on the THI cutoff score of 56, concerning the impact of tinnitus on the patient’s daily activities. In this regard, we had some risk avoidance for re-naming the low and high THI groups as low and high distressed groups, but our results came to support this classification. In our comparisons, we found significant differences between the high and low THI groups in the right OFC and DLPFC in alpha frequency band, which was in agreement with previous studies (Joos et al., 2012). The same is true for the beta frequency band differences in dACC (Song et al., 2013a) and the gamma band in the left DLPFC (Adamchic, Langguth, Hauptmann, & Tass, 2014). Other supportive findings were considered in the functional connectivity contrast between the two groups. Increased functional connectivity in the high THI group between the secondary auditory cortices and the frontal areas, and also between the parahippocampus and the centrofrontal regions is consistent with the previously reported sLORETA studies (Hassan, Dufor, Merlet, Berrou, & Wendling, 2014).

In conclusion, the THI score is a suitable tool for determining the tinnitus distress with a good relation to the EEG oscillatory alterations caused by tinnitus distress. Although such inference was formerly proved for TQ (Joos et al., 2012) and the latter is used widely as a distress indicator in tinnitus studies, THI questionnaire has fewer items and is more easily scored and clinically used (Kleinstäuber et al., 2015). Taken together, from our findings and depending on the others reports (Monzani et al., 2008; Newman et al., 1996; Salviati et al., 2013), we can conclude that THI is a convenient tool for use in diagnostic purposes, monitoring the effects of treatment and also for the sake of research. Lastly, this study had some limitations such as the small sample size since it is always better to include more participants, especially for EEG studies and correlation analysis. Also, it is better to analyze the subscales of THI (the functional, emotional and catastrophic) that may help reach the optimal use of the questionnaire.

## Ethical Considerations

### Compliance with ethical guidelines

The study procedure was according to the Declaration of Helsinki principles and the ethics committee of Iran University of Medical Sciences confirmed the study procedure (1395.9321667001, 22/4/2017).
